# Emerging Investigator Series: COVID-19 lockdown effects on aerosol particle size distributions in northern Italy†

**DOI:** 10.1039/d1ea00016k

**Published:** 2021-07-08

**Authors:** Jiali Shen, Alessandro Bigi, Angela Marinoni, Janne Lampilahti, Jenni Kontkanen, Giancarlo Ciarelli, Jean P. Putaud, Tuomo Nieminen, Markku Kulmala, Katrianne Lehtipalo, Federico Bianchi

**Affiliations:** Institute for Atmospheric and Earth System Research/Physics, Faculty of Science, University of Helsinki Finland federico.bianchi@helsinki.fi; Department of Engineering “Enzo Ferrari”, Università di Modena e Reggio Emilia Modena Italy; Institute of Atmospheric Sciences and Climate, National Research Council of Italy Bologna Italy; European Commission, Joint Research Centre (JRC) Ispra Italy; Aerosol and Haze Laboratory, Beijing Advanced Innovation Center for Soft Matter Science and Engineering, Beijing University of Chemical Technology Beijing China; Joint International Research Laboratory of Atmospheric and Earth System Sciences, School of Atmospheric Sciences, Nanjing University Nanjing China; Finnish Meteorological Institute Helsinki Finland; Institute for Atmospheric and Earth System Research/Forest Sciences, Faculty of Agriculture and Forestry, University of Helsinki Finland

## Abstract

The lockdown measures implemented to curb the COVID-19 epidemic in Italy reduced human mobility dramatically, which resulted in a marked decline in traffic intensity. In this study, we present the effect of lockdown measures on several air pollutants, particle number size distribution as well as on regional new particle formation (NPF) frequency in the Po Valley (northern Italy). The results show that during the lockdown period, concentrations of nitrogen dioxide (NO_2_), nitric oxide (NO), benzene (C_6_H_6_), and toluene (C_7_H_8_) decreased, while ozone (O_3_) concentrations mildly increased as compared to the corresponding period in 2016–2019. Unlike gaseous pollutants, particulate matter mass concentrations (PM_2.5_ and PM_10_) showed no significant changes. The impact of lockdown measures on particle number size distributions were also quite limited. During the lockdown period, the number concentrations of 10–25 and 25–50 nm primary particles were reduced by 66% and 34%, respectively, at the regional background site (Ispra) but surprisingly there was no difference during and after lockdown at the urban background site (Modena). Conversely, the NPF frequency was exceptionally high, 70%, in Modena during the lockdown as compared to values (22–26%) observed for the same period in 2006 and 2009, while NPF frequency in Ispra only slightly increased compared to the same period in 2016–2019. The particle growth rates, however, were slightly lower during the lockdown at both sites compared to other periods. The study shows that a drastic decrease in traffic had little influence on particulate pollution levels in the Po Valley, suggesting that other sources and processes also have a prominent impact on particle number and particulate matter mass concentration in this region.

## Abstract 2

Impact of lockdown measures on the air pollutants and particle number size distribution.

## Graphical abstract

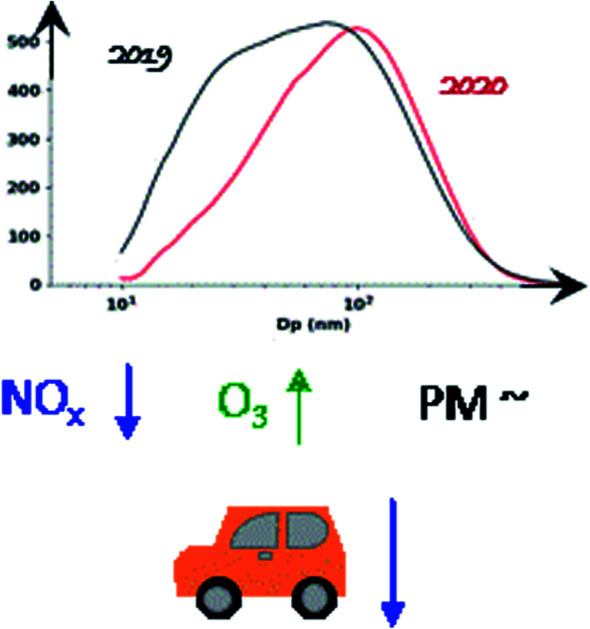

**Environmental significance**

The Po Valley is one of the most polluted areas in Europe due to traffic, intense activities of agriculture and factories and also due to a particular orography which favors accumulation of pollutants. The lockdown measures during the COVID-19 epidemic reduced the traffic intensity dramatically, which resulted in a mitigation of NO_*x*_ concentrations but had a limited impact on particulate matter mass concentrations. The effect of the lockdown measures on the particle number size distribution was subtle, reducing the number of primary sub-50 nm particles in the regional background site, and increasing the frequency of new particle formation mainly in the urban background site. This highlights the importance of emission sources other than traffic and of other processes for controlling particulate pollution in the Po Valley.

## Introduction

The outbreak of the coronavirus disease 2019 (COVID-19) caused a worldwide pandemic in early 2020, with rising global death tolls through widespread human transmission.^1^ Countries enforced restrictions to control and prevent the COVID-19 epidemic, like the shutdown of schools and restaurants as well as remote working. These implementations curtailed the emissions from motor vehicle traffic dramatically in many regions.^2,3^ Anthropogenic emissions from human activities influence air quality, but separating and measuring the different impacts of various activities is challenging because they are simultaneous and mixed together. Therefore, these lockdown episodes offered a unique opportunity to observe possible changes in air pollution due to the sudden and unusual reduction in the traffic intensity.

Previous research on COVID-19 restrictions has focused on common air pollutants such as NO_2_, NO, O_3_, PM_2.5_, and PM_10_ to investigate air quality during lockdown periods in many geographic areas including China and Europe.^2–5^ Preliminary observations have revealed expected changes in the concentrations of some of these pollutants, but also some unexpected behaviours. As expected, NO_2_, NO, PM_2.5_, and PM_10_ concentrations dropped when traffic was drastically reduced over several areas around the globe. For example, during the lockdown periods, the average NO_2_ column drop over Chinese cities amounted to 40%^6^ compared to the same period in 2019, and its decrease in western Europe and the United States was also significant (20%–38% (ref. 3)) when compared to the same period in 2019. Surface *in situ* measurements also indicated a reduction in PM_2.5_ concentrations in South Korea (54%), Los Angeles (31%), and in southern and central China (10% to 60%) compared to the same period in 2019.^2,3,7^ However, unexpected increases in PM_2.5_ concentrations occurred in northern and eastern China,^2,4^ which are known to experience some of the worst air qualities in the world. Observations and model simulations^2^ indicated that the reduced NO_*x*_ (nitrogen oxides) emissions enhanced ozone concentrations, which further increased the atmospheric oxidizing capacity and therefore the formation of secondary aerosol. However, studies about the effect of lockdowns on the particle size distribution are still limited.

The Po Valley, located in northern Italy, is one of the most critical regions for air pollution in Europe because of its particular orography^8^ and the presence of numerous anthropogenic activities including intensive livestock farming and agriculture, road traffic, and factories. A lot of effort has been put during the last years to understand air pollution characteristics of this region, including the chemical composition of aerosol particles,^9–11^ and the formation and growth of new particles.^12–14^ A source apportionment study from Larsen *et al.*^15^ indicates that secondary aerosol was the dominating source (54–75%) and that traffic (16–17%) and biomass burning (10–12%) were the major primary sources of aerosol mass across the Po Valley during the winters of 2006–2009. The strict lockdown period for the Po Valley area lasted from 8 March to 4 May 2020. The surface measurements performed in Lombardy^16^ indicated that NO_2_ concentrations decreased by 30% and 40% on average and that PM_10_ concentrations were not significantly affected in urban and regional background sites in this region.

The emissions from traffic contribute to particulate matter in the urban environment and pose a significant risk to human health by increasing premature mortality.^17^ Traffic is a major source of atmospheric nanocluster aerosols (particles smaller than 3 nm), and it directly emits aerosol particles including the core mode (particles smaller than 10 nm) and soot mode (particles between 30 and 100 nm in diameter),^18–20^ what we call primary aerosol particles. In addition, traffic emissions have been shown to significantly produce sub-3 nm particles, and also to contribute to secondary aerosol particle formation by emitting various aerosol precursors,^21,22^ including SO_2_ (sulfur dioxide), VOCs (volatile organic compounds) and NO_*x*_. The traffic sector is considered as one of the major sources of NO_*x*_ emission,^23,24^ which can affect O_3_ concentration by O_3_ titration with NO and O_3_ nonlinear formation chemistry. In the atmosphere, the oxidation of VOCs by O_3_ and SO_2_ by OH radicals form low-volatility compounds like highly oxygenated compounds (HOMs)^25,26^ and sulfuric acid (H_2_SO_4_), which contribute to formation and growth of new aerosol particles.^25,45^ Therefore, reduced traffic intensity can affect the atmospheric oxidizing capacity and consequently the formation of HOMs and H_2_SO_4_. Furthermore, reduced particle emissions may also lower the condensation sink of these low volatile vapors, favoring new particle formation. However, it is not clear how the restrictions affected new particle formation as well as SOA (secondary organic aerosol) formation and, consequently, to what extent particle size distributions were modified by traffic reduction during the lockdown period.

In this study, we compared the air pollutants (PM_2.5_, PM_10_, NO_2_, NO, O_3_, benzene, and toluene) during the lockdown period to the same time period in 2016–2019 for five cities of Modena, Bologna, Reggio Emilia, Parma, and Piacenza. Additionally, we explored the changes in particle number size distributions in Modena, and at a rural background site in the Po Valley (the European Commission atmospheric observatory in Ispra). Particle number size distribution in the 2–42 nm size ranges measured at the urban site in Modena during and after the lockdown were analysed in light of traffic data. This analysis was supported by the long term (2016–2020) particle number size distribution (10–800 nm) data from the regional background site in Ispra. The impact of traffic reduction on the concentration of primary particles smaller than 50 nm was addressed. Regarding new particle formation, we studied its monthly frequency and compared the lockdown period to previous years in both sites. Thereby, this study aims to increase the understanding of the impact of lockdown measures on particle number size distributions in the Po Valley, including primary ultrafine particle emissions and new particle formation frequency.

## Methods

### Measurement data

The atmospheric composition data analysed in this study include data collected from the regional air quality monitoring networks, observations conducted by the ACTRIS (Aerosol, Clouds and Trace Gases) Research Infrastructure and data from an *ad hoc* deployment of aerosol samplers with a focus on the Po Valley area. The air quality data include air pollutants such as PM_2.5_, PM_10_, NO, NO_2_, O_3_, as well as benzene, and toluene. Data from 12 January 2016 through 5 July 2020 were made available by the provinces of Bologna, Modena, Parma, Piacenza and Reggio Emilia (Table 1) by the Emilia-Romagna Regional Environmental Agency^27^ using sampling equipment following a quality management system, which is certified with ISO 9001:2008. These data have been automatically and manually validated by the Agency and undergo a daily, seasonal, and annual comparison with nearby sites.

**Table 1:** The measurement data and its origin^a^

	Bologna	Modena	Parma	Piacenza	Reggio Emilia	Ispra
NO_2_, NO, PM_2.5_, PM_10_	Ub, Ut	Ub, Ut	Ub, Ut	Ub, Ut	Ub, Ut	
O_3_	Ub	Ub	Ub	Ub	Ub	
C_6_H_6_, C_7_H_8_	Ut	Ut	Ut	Ut	Ut	
Particle size distribution		Modena station				JRC-Ispra observatory
Traffic vehicle count		Whole city				

a Ub refers to the urban background sites, Ut refers to the urban traffic sites. Urban background sites represent locations not significantly influenced by a local source, while the traffic sites refer to the locations where the pollution level is determined predominantly by the emissions from the nearby traffic. See Fig. S1 and S2 (see ESI) for a map of the measurement locations.

The number size distribution of neutral and charged particles was measured with a Neutral cluster and Air Ion Spectrometer (NAIS, Airel Ltd., Estonia^28^) deployed at the grounds of the University of Modena and Reggio Emilia (44°37′ N, 10°57′ E; 34 m a.s.l.) *ca.* 10 meters above the ground. The site is located in a pedestrian area and is classified as urban background, with the two major streets at about 200 m and 270 m away. The NAIS provides the number size distribution of ions and total (charged and neutral) particles with electrical mobilities between 3.2 and 0.0013 cm^2^ V^−1^ s^−1^ which corresponds to 0.8–42 nm in mobility diameter, divided in 21 mobility bins.^28,29^ In the total particle measurement mode, only the data between *ca.* 2 and 42 nm can be used, due to the ions produced in the corona charger of the instrument which affect the particles below 2 nm. The data, with a time resolution of 90 seconds, was collected from 30 March 2020 specifically for this study. In this manuscript we focus on the total particle size distribution data since it is more important when investigating the particle emissions.

Particle number size distribution from the observatory of the European Commission – Joint Research Centre of Ispra (45°48′ N, 8°38′ E; 217 m a.s.l., hereafter IPR) were provided by the Global Atmospheric Watch – World Data Centre on Aerosols.^30^ The data was obtained from a Differential Mobility Particle Sizer (DMPS) and includes particle number concentration between 10 nm and 800 nm with 45 mobility bins from January to June in 2016–2020 with a 12 minute time resolution. IPR is a regional station within the GAW network where ACTRIS quality assurance/quality control procedures are followed for the DMPS measurements as described in Laj *et al.*^31^ IPR is classified as a rural background station for the Po valley,^32^ given its distance of tens of kilometres from major local pollution sources (Fig. S1†).

**Fig. 1:**
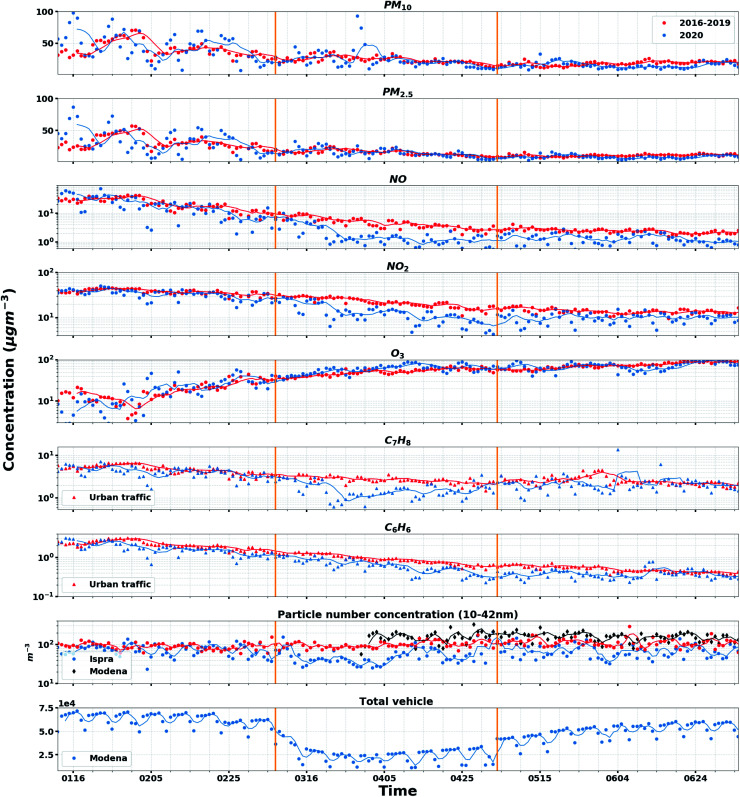
Time evolution of the daily average particulate matter (PM_2.5_, PM_10_) concentrations, trace gas (NO_*x*_, O_3_) for all urban background sites, benzene (C_6_H_6_) and toluene (C_7_H_8_) for urban traffic sites, daily averages of 10–42 nm particle number concentration (Ispra and Modena), and vehicle count (Modena). The red dots indicate the mean values from the daily average of 2016–2019, and their moving average is shown with the red lines, whereas the blue and black dots and blue and black lines indicate the values for the year 2020. The period between the two orange vertical lines represents the lockdown period in Italy.

Direct traffic counts were available in Modena from 1 January 2019 until 31 July 2020: these data were collected by 400 induction loops for traffic light control within the urban and suburban street network. Vehicle counts from all induction loops were aggregated into one hour traffic count across the whole urban area (Fig. 1). One hour counts were also computed for the loops at the crossroad few tens of meters away from the air quality monitoring station representative of the urban traffic conditions in Modena. The traffic vehicle count from the Via Vignolese induction loops, one of the closest streets to the NAIS measurement station (see in Fig. S2†), was calculated separately.

Finally, meteorological variables in Modena were provided by the monitoring station at the campus of the University of Modena and Reggio Emilia (nearby the NAIS instrument) and included wind direction and speed at 10 meters above ground.

### Data analysis

We classified the days in Modena into three different categories regarding the occurence of regional new particle formation (NPF) events: (1) event days, (2) undefined days, (3) and non-event days. This was done by evaluating the particle size distribution data from the NAIS in the Modena station and from the DMPS at the JRC-Ispra observatory following the method presented by Dal Maso *et al.*,^33^ and Hirsikko *et al.*^34^ Examples of the three categories are shown in Fig. S4.† The regional NPF event days are characterized by a sudden increase in the concentration of particles below 25 nm, and a continuous growth of the new particle mode lasting for several hours. The days without appearance and growth of particles below 25 nm were classified as non-event days. The remaining days when particle bursts where observed, but it was not clear whether they were connected to regional NPF, were classified as undefined days. In Modena, most new particle formation events show patterns different from the normal ‘banana’ shape, which is characteristic for cleaner environments, *e.g.* boreal forests. As shown in Fig. S6a,† the burst of nanometer-sized particles (<3 nm) lasts for several hours. Therefore, the appearance time^35^ was used to retrieve the growth rates of the <3 nm, 3–7 nm, and 7–20 nm particles for the identified NPF events in the Modena station. In the appearance time method, we retrieve for each particle size bin the time when the concentration reaches 50% of its maximum value.

The DMPS measured particle number size distributions at IPR were used to calculate the condensation sinks for 2016–2020. The condensation sink describes how fast condensable vapors are lost due to condensation on the aerosol population and it depends on the particle size distribution. In this study, we calculate the condensation sink according to Pirjola *et al.*^36^

In case of the NPF events at IPR, the particle growth rate was calculated using the maximum concentration method,^37^ which is much better when normal ‘banana’ shapes are observed. In the maximum concentration method, the times when the concentration is at the maximum in each size bin are determined. The growth rates in these two methods were obtained as the slope of the linear fit of the times with the corresponding geometric mean diameters of the particles.

As shown in Fig. 1, we calculated the daily average concentrations for all pollutants at all sites for the period 12 January–5 July from 2016 to 2020, which comprises the 8 lockdown weeks (9 March–3 May), the 8 weeks before the lockdown (12 January–8 March), and 8 weeks after the lockdown (4 May–5 July). The data are classified into urban background and urban traffic. The statistical significance of the differences in daily average values during the lockdown period in comparison with the mean values of previous years was assessed by applying a *t*-test assuming unequal variances between 2016–2019 and 2020 averages. The null hypotheses were tested at the 99% confidence level, and results were used to determine if differences between 2016–2019 and 2020 were statistically significant. The same method was also applied to particle number concentrations, condensation sinks, and growth rates. The statistics are listed in Tables S1–S4.†

## Results

### Impact of the mobility restrictions on air quality

The time series of the total vehicle counts in Modena is used here as a proxy for the mobility restrictions applied in the whole Po Valley area. Specifically, it decreased from 6.8 × 10^4^ h^−1^ (pre lockdown) to 2.6 × 10^4^ h^−1^ (lockdown period), corresponding to a −62% fractional change (Fig. 1).

Fig. 1 shows statistically significant (99% confidence level) decreases in NO_2_, NO, benzene, and toluene concentrations at the urban sites during the lockdown period compared to the previous years. NO_2_ concentrations exhibited the highest reduction fifteen days after the total vehicle counts started to decrease at the urban background sites (Fig. 1), and five days later at the urban traffic sites (Fig. S3†), while NO concentrations had a faster response to the vehicle number decline, with a five-day delay at both urban background and traffic sites (Fig. 1 and S3†). Conversely, O_3_ concentration shows a mild but statistically significant increase, likely due to the reduction of titration by NO.^4,38,39^

Unlike gaseous pollutants, 2020 levels of PM_2.5_ and PM_10_ mass concentrations are not different (on a 99% confidence level) from the 2016–2019 averages, which decrease from winter to summer, likely because of changes in residential heating emissions^40^ and in meteorological conditions. Instead, the 10–42 nm particle concentration at Modena station keeps essentially constant for the whole period of 30 March to 5 July. In contrast, the daily average number concentration of 10–42 nm particles at JRC-Ispra observatory drops and closely traces traffic change in March at the beginning of the lockdown period compared to the same time in 2016–2019. However, after 5 April, particle number concentrations start increasing again and fluctuating. The impact of primary particle emissions from traffic and NPF on number size distributions is discussed in next sections.

**Fig. 2:**
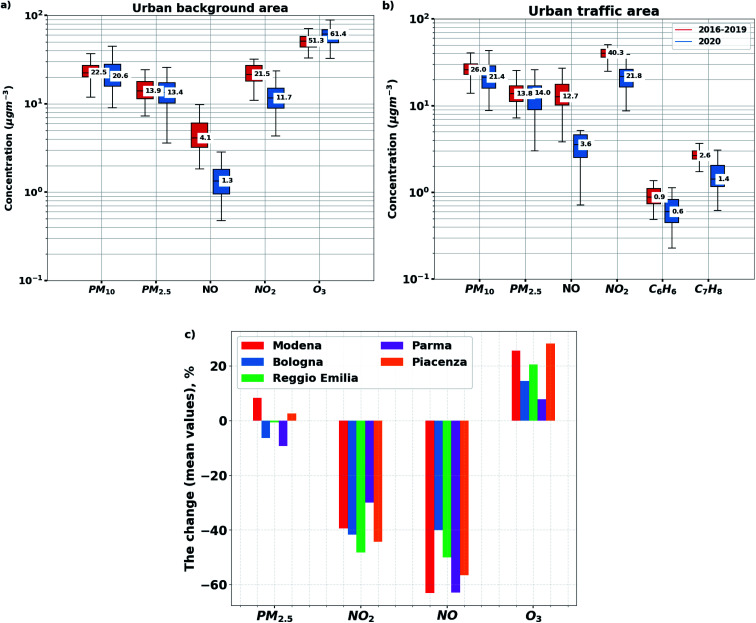
Trace gas and particulate matter concentrations for all urban background (a) and traffic sites (b) in the Po valley area during the lockdown period in 2020 (blue) and the same period in the years 2016–2019 (red). The boxes in (a) and (b) present the 25–75% of values, and the upper and lower whiskers refer to the lower and upper 5% of values. Panel (c) presents the change in air pollutants' concentrations for five cities.

As shown in Fig. 2a and b, reductions of 9.1 μg m^−3^ (−41%) and 2.8 μg m^−3^ (−59%) in NO_2_ and NO concentrations were observed, respectively, when compared to the mean level of previous years (2016–2019) at the urban background sites. The percentage decrease at the urban traffic sites was in the same range, *i.e.* 18 μg m^−3^ (−45%) and 9 μg m^−3^ (−66%) for NO_2_ and NO, respectively. Additionally, benzene and toluene concentrations dropped by 0.29 μg m^−3^ (−33%) and 1.1 μg m^−3^ (−37%). Ozone concentrations, however, increased by 8.8 μg m^−3^ (+18%) whereas, PM_2.5_ and PM_10_ present only slight changes since the difference between 2016–2019 and 2020 is not statistically significant (Fig. 2), suggesting that decreasing emissions from traffic did not result in a reduction of particulate matter concentrations. A detailed analysis using the Comprehensive Air-quality Model with eXtensions (CAMx) where anthropogenic vehicular emissions were reduced according to the mobility data presented here, supported similar conclusions for the changes in NO_2_, O_3_, and PM_2.5_ concentrations^41^ in the Po Valley. Here we have assumed that any anomaly in the meteorological conditions in year 2020 compared to the reference period had a minor impact compared to the reduction of traffic.

As shown in Fig. 2c, all cities reported reductions in NO_*x*_ levels and an increase in O_3_ concentrations, whereas changes in PM concentrations, although small, were either positive or negative depending on the site. For example, during the lockdown period, the average level of PM_2.5_ concentrations decreased by 9% in Parma, while it increased by 8% in Modena, indicating the complexity of PM concentration response to lockdown measures.

### The impact of the mobility restrictions on particle number size distributions

The shape of the particle number size distributions and particularly the number concentrations of particles smaller than 50 nm result from particle primary emissions, secondary particle formation, and different aerosol dynamic processes altering the size distribution. In addition to the surface measurements of the trace gas species, we also explored the changes in particle number size distributions during the lockdown. The analysis was based on particle number size distribution data from two sites: (i) a four-months NAIS data set available from the Modena station with measurements from the beginning of April 2020, and (ii) the long-term DMPS data from the JRC-Ispra observatory (2016–2020 data).

### Primary particle emissions from traffic

In the absence of regional NPF, particles below 50 nm, and even below 10 nm mainly come from traffic emissions, as shown in previous studies^19–22,42^ and to a certain extent corroborated by this study.

**Fig. 3:**
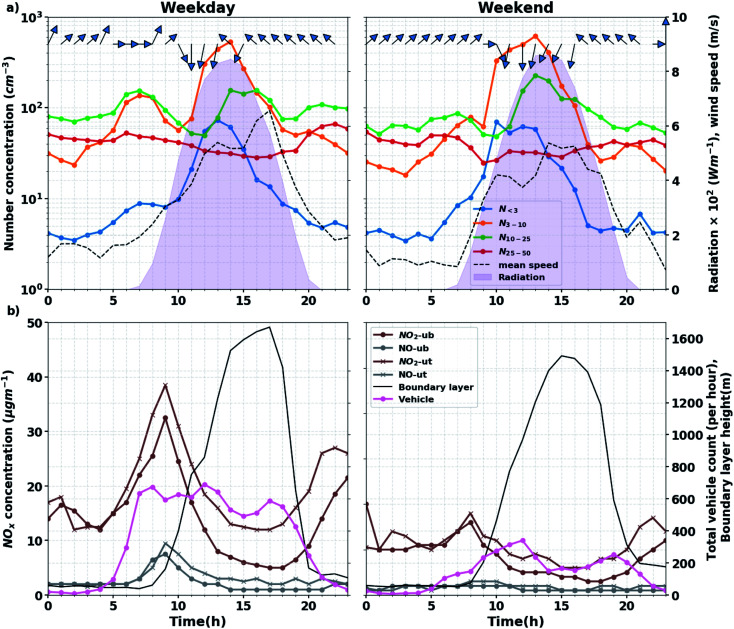
(a) Median diel variations during the lockdown period for particles <50 nm, solar radiation (shaded area), wind speed (black dash line) and direction (blue arrows). (b) NO, NO_2_ concentration from urban background (circle) and traffic (*x*) sites, total vehicle count (from the closest sensor) and boundary layer height.

Evidence can be found when looking at the diurnal variations of the 2–42 nm particle concentrations, NO_*x*_ concentrations, and total vehicle count at Modena station from 1 April to 3 May 2020 for in Fig. 3. On weekdays, the vehicle counts from the closest traffic sensors (800 m west of the measurement station see Fig. S2†) increases in the early morning, reaches the highest point at around 8:00 am and shows two additional peaks around noon (12:00) and at the afternoon rush hours (17:00). On weekends, the total vehicle count is lower compared to weekdays, and progressively increases from the early morning to reach a maximum at noontime (12:00). The impact of traffic on air pollution is illustrated by the diel variations of NO_2_ and NO concentrations tracing those of the total vehicle count during the morning rush hours on weekdays, and by the daily mean NO_2_ and NO concentrations being respectively 1.7 and 4.3 times higher during weekdays than during weekends. Before sunrise (when no NPF is expected), particle number concentrations are also greater during weekdays than during weekends in three size bins (3–10, 10–25 and 25–42 nm). Furthermore, the concentration of particles in the size ranges between 2 and 42 nm closely traces the traffic intensity during the morning rush hours on weekdays.

**Fig. 4:**
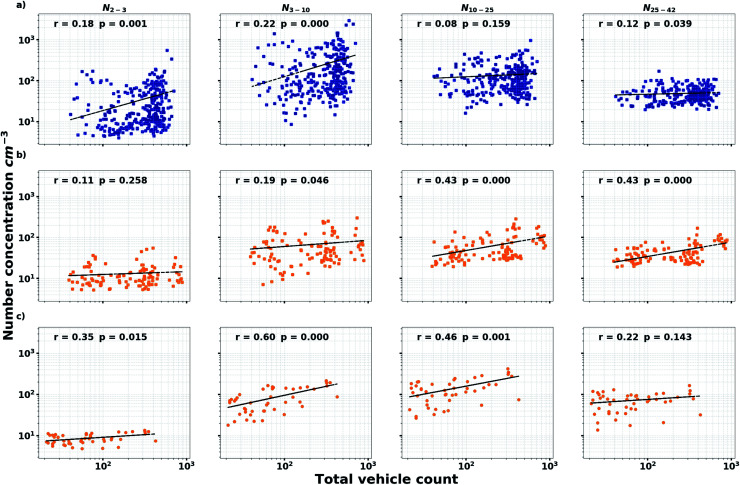
Correlation between the total vehicle count and the particle concentration in different size bins during the lockdown period (12 January–8 March) in Modena for (a) NPF event days during daytime (7–20); (b) non-NPF event days during daytime; (c) non-NPF event days during nighttime (21–6).

The importance of traffic on nanoparticle concentration is greatest in the absence of other sources, like regional NPF. The correlation between the concentration of 10–42 nm particles and traffic counts are better during non-event days than during NPF days (Fig. 4). For sub-10 nm particles, the correlation is better during nighttime than daytime. Particle concentrations are of course also affected by meteorological conditions controlling turbulence, convection, and advection. Therefore, we excluded cases when the boundary layer got shallower a few hours before sunrise, affecting the concentrations as seen in Fig. S4,† but we still observe the early morning peak for 3–10 nm particle before sunrise on weekends.

After the lockdown, the total vehicle count increased by a factor of 2 in Modena (Fig. S5†). NO and NO_2_ concentrations increased during the morning rush hours by a factor of ∼2 and ∼1.3, respectively, at both the traffic and urban sites in Modena. However, the primary particle concentrations in the morning rush hours (before regional NPF occurred) did not increase (compared to the lockdown period) at the NAIS measurement site. Selecting days on which non-zero westerly winds were observed after the lockdown as during the lockdown, and with similar mixed boundary layer heights, shows that meteorology could not be the cause for this absence of increase in primary particle number concentration after the lockdown. Therefore, the morning peak in primary particle concentration seemed to be connected to traffic when comparing the week days and weekends during the lockdown, but not when comparing week days during lockdown and after lockdown. We could not find any plausible explanation to this observation so far.

**Fig. 5:**
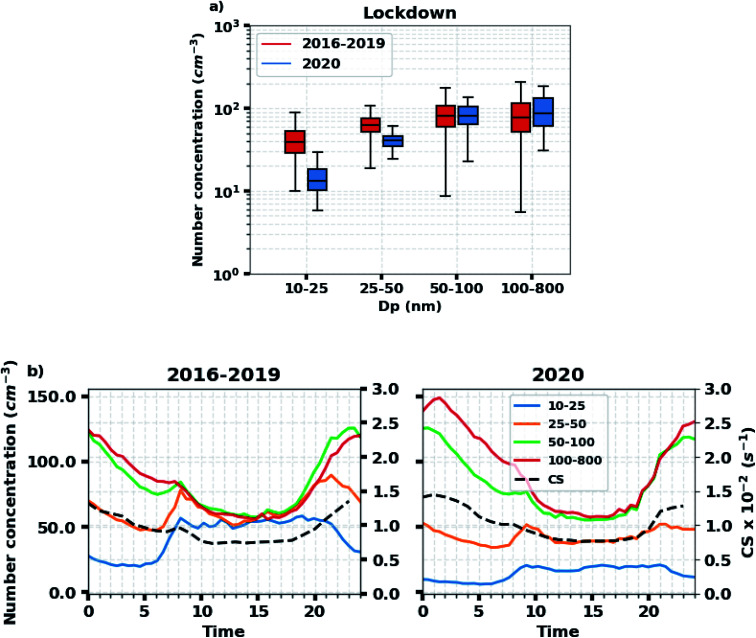
Particle concentrations in various size bins derived from the DMPS measurements at the Ispra site. (a) The particle concentrations during the lockdown period in 2020 (blue) and for the same period in 2016–2019 (red). (b) Diel variations of the particle concentrations and condensation sink for the lockdown period in 2020 (right) and for the same period in 2016–2019 (left). Note: all the regional NPF event days are excluded from this plot to better see the effect of traffic reduction.

Fig. 5 shows mean particle concentrations in various size bins at JRC-Ispra observatory for days where no regional NPF events were detected. During the lockdown period, the 10–25 and 25–50 nm particle number concentrations were in 2020 significantly (99% confidence level) lower than that in 2016–2019 (Fig. 5a): reduction of 66% and 34% were observed for the mean values of the 10–25 and 25–50 nm particle number concentrations, respectively. Conversely, the difference between 2020 and 2016–2019 in the concentrations of particles larger than 50 nm was not statistically significant. Therefore, the fraction of sub-50 nm particles was lower during the lockdown period than during the corresponding period in 2016–2019. To explore this further, we looked at the diel variations of the particle number concentrations in 4 different size bins. As shown in Fig. 5b, the 10–25 and 25–50 nm particle number concentrations in 2020 were much lower compared to their concentrations in 2016–2019 during the whole day. The morning rush hour peak for the 10–50 nm particles in 2016–2019 appeared around 8:00 am, and was much greater than that (at 9:00 am) in 2020. In contrast, the diel variations in the number concentrations of particles in the size bins 50–100 and 100–800 nm were remarkably similar during the lockdown period in 2020 compared to 2016–2019. Likewise, the diel variations of condensation sink, which closely trace the 50–800 nm particle concentration, were very similar in 2016–2019 and 2020, ranging from 0.007 to 0.015 s^−1^ across the day. This indicates that the decline in 10–50 nm primary particle concentrations at JRC-Ispra observatory during the lockdown was not caused by an increased sink due to larger particles.

The correlation between the number concentrations of particles in size bins ranging from <3 to 50 nm and traffic intensity (on non-NPF days) suggest that traffic is a significant source of <50 nm primary particles at both measurement sites. Lower concentrations of <50 nm particles during the lockdown period at the regional background site in Ispra was most likely caused by decline of the traffic due to the mobility restriction measures. The lack of increase in primary particle number concentration after the lockdown at the urban background site Modena is all the more surprising, showing the complexity of the sources and processes related to atmospheric aerosols.

### New particle formation and growth

The NAIS at the Modena station was operated from April to September 2020 continuously for about 241 days. Fig. 7a shows the monthly distribution of regional NPF event days, non-event days and undefined days for April–June. In general, both the frequencies of NPF event days and non-event days decreased from April to June 2020, while the number of undefined days increased. However, the number of NPF event days in April 2020 during the lockdown (23 days) was not much different from the number of NPF event days in May–June (22 and 21 days, respectively), while the difference in traffic counts was about a factor of 2. The comparison of the diel cycles observed during weekdays and weekends confirm that a decrease in traffic intensity does not systematically lead to a decrease in NPF. On the contrary, Fig. 3 shows that the number of <3, 3–10, and 10–25 nm particles appearing when solar radiation increases is slightly greater during weekends than during week days. This is consistent with the fact that on NPF days, traffic intensity and particle concentrations in different size classes show only marginal correlation (*r* < 0.22) during daytime (Fig. 4a). Actually, looking into the historical data from Modena station (Fig. 7b), the fraction of NPF days for the 1 April–3 May 2020 period during the lockdown (70%) was much higher than that for the same period in 2007 and 2009 (22–26%). In addition, the NPF frequencies observed at a rural background observation station^12^ located 60 km to the west of Modena in the past (April–May 2002–2005) suggest that low traffic conditions (as encountered during the lockdown period) favor NPF.^43^

The long-term measurement of the aerosol size distribution at the JRC-Ispra observatory makes it possible to compare the monthly fraction of NPF days in 2020 with previous years. We analyzed the data from January to July for years 2016 to 2020 (Fig. 7c). During this period, the DMPS instrument was operational on 968 days (91% of all the days). In general, regional NPF events occur on 0–36% of the days, and their monthly frequency generally increases from January to July. However, the maximum NPF events frequency occurs between April and July, depending on the year. The number of NPF days in April 2020 during the lockdown (10 days, 33%) was higher than during previous years (10–27%), while in May 2020 (after the lockdown) it was comparable to 2016–2019. It was similar to 2016–2019 also in March 2020 (during the lockdown), but the large inter-annual variability in the NPF event occurrence in March suggests a big influence of weather conditions on NPF during that particular month. Therefore, the lockdown measures could have had a slight impact on the occurrence of NPF at IPR too.

As already discussed, the enhancement of O_3_ concentrations observed during the lockdown period in NO_*x*_-rich areas can further increase the atmospheric oxidizing capacity and, therefore, enhance the production of condensable vapors such as H_2_SO_4_ and HOMs.^4^ Furthermore, the NPF suppression effect caused by NO (because NO reduces the formation of HOM dimers^44,45^) should decrease with the reduction of NO emissions. Therefore, the lockdown measures could well have led to an increase in NPF event frequency, given that the emissions of the main precursors (SO_2_ and/or VOCs) did not decrease substantially. The occurrence of NPF is likely affected also by ammonia or amines, which serve as a base for stabilizing the initial clusters.^46,47^ The agriculture activities in the Po Valley lead to a high amount of ammonia and amine emissions into the atmosphere,^48^ and they continued as usual during the lockdown period.

**Fig. 6:**
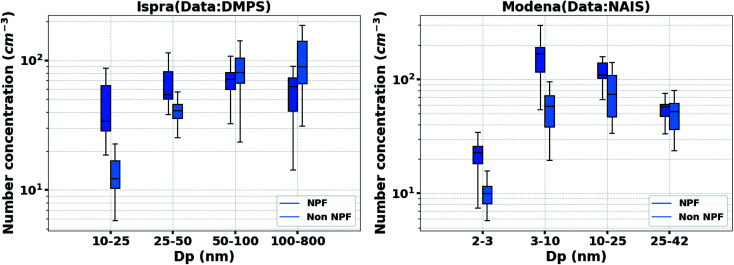
Particle number concentration during the lockdown period at JRC-Ispra (DMPS) and Modena stations (NAIS). The dark blue boxes refer to the 25–75% of the daily mean value of the particle number concentrations on NPF days, while the light blue boxes refer to the data from non-NPF days. The upper and lower whiskers refer to the lower and upper 5% of values. Note the different *X* axis.

As shown in Fig. 6, regional new particle formation (NPF) events significantly increased the particle number concentrations, especially those of small particles in the size range of 2–25 nm at the Modena station and 10–50 nm at JRC-Ispra observatory, also during lockdown. Therefore, NPF could have partially counterbalanced the reduction of <50 nm particle emission from traffic during the lockdown in Ispra, and contributed to the sustained <50 nm particle concentrations during the lockdown in Modena.

**Fig. 7:**
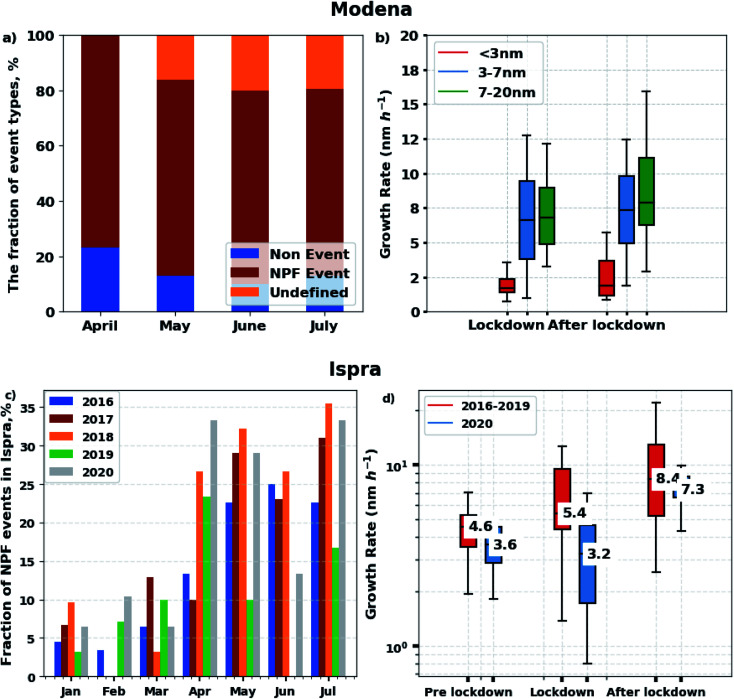
NPF frequencies and particle growth rate on regional NPF event days at the Modena and JRC-Ispra sites. (a) Monthly fraction of NPF event days, non-events and undefined days at Modena station in 2020; (b) the growth rate from NAIS at the Modena station; (c) monthly fractions of NPF event days at the JRC-Ispra observatory for January–June from 2016 to 2020 (no data from 31 May to 19 July 2019); (d) the growth rate for the particles in the size range of 10–20 nm from DMPS at the JRC-Ispra observatory.

However, all data indicate that the particle growth rate (GR) was reduced during the lockdown compared to other periods. In Modena, the GR of <3, 3–7 and 7–20 nm particles were on average slightly lower during the lockdown than after the lockdown (Fig. 7b). In Ispra, the GR of 10–20 nm particles (Fig. 7d) decreased during the lockdown period in 2020 (average 3.2 nm h^−1^), both compared to before (3.6 nm h^−1^) and afterwards (7.3 nm h^−1^), and to the 2016–2019 average across the 9 March–3 May period (5.4 nm h^−1^). The reduction of growth rate during the lockdown period in 2020 might be explained by the decrease in the condensable species contributing to particle growth due to the lockdown measures. Many earlier observations show that a different set of vapors are responsible for the first steps of NPF and the further growth of the formed particles (*e.g.* Kulmala *et al.* 2013 (ref. 49)), and therefore it is possible that the NPF occurance increases, but the forming particles grow slower. For example, the oxidation products of aromatic VOCs, which originate *e.g.* from traffic, can contribute to particle growth, while their importance to NPF is small.^44,50^ These observations challenge observation and modelling data^2,4^ which show that the abatement in NO_*x*_ emission during the lockdown period led to an increase in secondary aerosol formation.

## Conclusions

High NO_*x*_ and PM_2.5_ concentrations are often affecting the entire Po Valley region due to intense road traffic, livestock farming, fertiliser spreading, industrial activities, and the particular meteorology and orography of this region. The traffic sector is considered as one of the major sources of particulate matter in the Po Valley as indicated by numerous source apportionment studies.^15^ The lockdown measures for controlling the spread of the COVID-19 in spring 2020 reduced the traffic intensity dramatically in northern Italy, which can serve as a benchmarking experiment to evaluate air-quality responses to a sharp traffic emission reduction in this region.

A strict lockdown took place from 9 March to 3 May 2020 in Italy. During this lockdown period, urban traffic was largely reduced (*e.g.* −60% in Modena). Across five large cities in the Po Valley, a large reduction in NO, NO_2_, benzene, and toluene concentrations, as well as a mild but statistically significant increase in O_3_ concentrations could be observed by a comparison with the corresponding period in 2016–2019. The increase in O_3_ concentration was likely due to the reduced titration of O_3_ by NO in urban areas. Unlike gaseous pollutants, mean particulate matter concentrations (PM_2.5_ and PM_10_) were not significantly different during the lockdown period in 2020 as compared to the 2016–2019 average.

The impact of the lockdown measures on particle number concentrations were significant for primary particles with diameters of 10–25 nm (−66%) and 25–50 nm (−34%) at the regional background site (Ispra) comparing the 2020 values to the average of 2016–2019 on non-event days. However, at the urban background site (Modena), where we started the measurements of 2–42 nm particles during the lockdown in 2020, we could not see a clear increase in the sub-50 nm concentrations after the traffic returned to normal levels. As for regional new particle formation, NPF events occurred on 70% of the days at the Modena station during the lockdown period, *i.e.* much more frequently than in the same period in 2007 and 2009 (22–26%). At the JRC-Ispra site, a slight increase in NPF occurrences was also observed in April 2020 (during the lockdown) compared to previous years (2016–2019), but the year-to-year variation is significant. On the other hand, the particle growth rate was reduced during the lockdown compared to other periods in both Modena and Ispra, which might be related to the enforced measures reducing the emission of the gaseous precursors of condensable species. However, the reduction of the growth rate during the lockdown period in 2020 does not support the increase in secondary aerosol formation arising from lockdown measures suggested by other studies.^2,4^ Additional measurements, including the concentrations of the condensable vapors, would be of great help for future studies.

The limited impact of the lockdown measures on particle number size distribution is consistent with the lack of systematic decrease in PM mass concentration observed in various cities across northern Italy. These observations do not imply that road traffic does not significantly contribute to particulate pollution. They could also be explained by the compensation of reduced traffic emission reductions during the lockdown by other processes, including increased emission from other sectors (*e.g.* domestic heating), increased formation of secondary aerosol due to an increased oxidizing capacity of the atmosphere, or decreases in particle sinks related to unusual meteorological conditions.

## Data availability

Measurements data will be made openly available before the final publication.

## Author contributions

F. B. designed the study. J. S. led the work, performed the data analysis, and wrote the paper. A.B. prepared the raw data of the air pollutants, maintained the DMSP, traffic intensity and wrote the method. A. B., A. M., and J. L. installed and maintained the NAIS. F. B., A. M., J. K., G. C., J. P. P., M. K., K. L. and T. N. helped with the interpretation of the results and recommendation for the analysis. All the authors commented on the manuscript.

## Conflicts of interest

The authors declare no competing interests.

## Supplementary Material

EA-001-D1EA00016K-s001
