# Supplementary material for: Emerging Investigator Series: COVID-19 lockdown effects on aerosol particle size distributions in northern Italy
Source: Environ Sci Atmos. 2021 Jul 8;1(5):214–27. doi: 10.1039/d1ea00016k (PMC8296575; doi:10.1039/d1ea00016k)
Supplement: EA-001-D1EA00016K-s001 [file EA-001-D1EA00016K-s001.pdf]

Electronic Supplementary Material (ESI) for Environmental Science: Atmospheres.
This journal is © The Royal Society of Chemistry 2021

Supplementary material

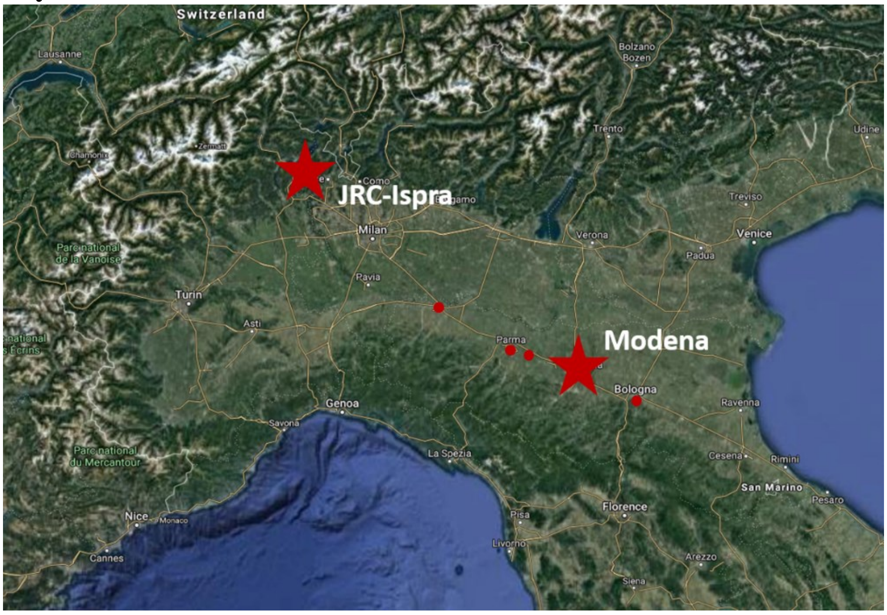

Figure S 1. The location of the JRC-Ispra (45.82 N, 8.64E) and Modena (44°37' N, 10°57' E) sites. And other cities with red rots.

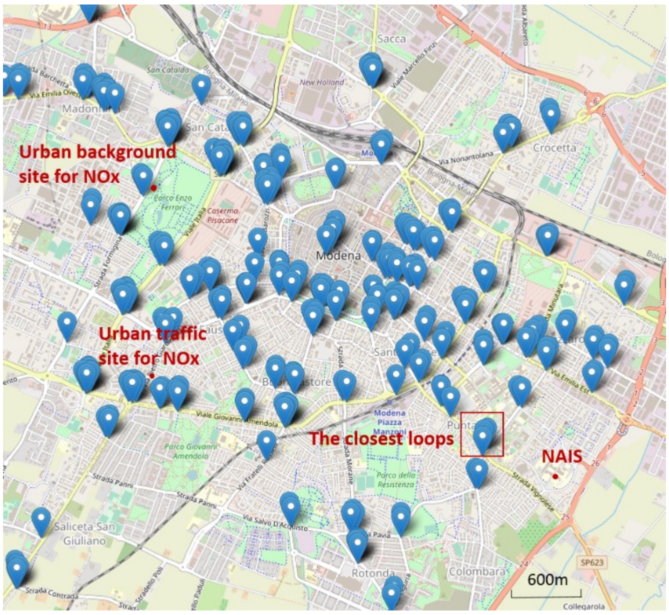

Figure S 2. The location of the NAIS measurements and the traffic count loops (blue marker) in Modena.

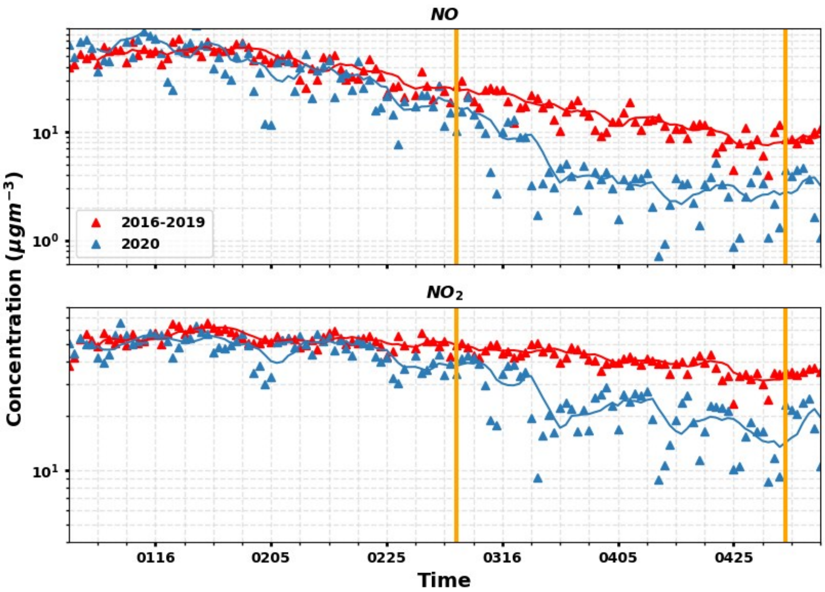

Figure S 3. Time evolution of the daily mean NO (upper panel) and NO_2 (lower panel) concentrations in the urban traffic sites during pre and lockdown period (8 Mar – 4 May) in 2016-2019 (in red) and 2020 (in blue).

Table S 1. The statistical difference between gas/particle concentrations in 2020 and 2016-2019

	t-stastistic				P-value			
	Ubran backgroud	Urban traffic	Fig 5a	Fig 5b	Ubran backgroud	Urban traffic	Fig 5a	Fig 5b
NO	7.8	9.9			3.6×10^{-12}	3.6×10^{-17}		
NO_2	8.2	12.4			4.0×10^{-13}	4.8×10^{-23}		
$PM_{2.5}$	0.1	0.7			0.9	0.5		
PM_{10}	0.08	0.7			0.9	0.5		
O_3	3.9				1.5×10^{-4}			
C_6H_6		6.3				6.7×10^{-9}		
C_7H_8		8.9				1.1×10^{-14}		
10-25nm			8.1	12.9			3.7×10^{-14}	1.6×10^{-22}
25-50nm			6.3	11.3			1.8×10^{-9}	2.8×10^{-19}
50-100nm			0.2	0.1			8.6×10^{-1}	0.89
100-800nm			1.6	2.11			0.12	0.04

Table S 2. The statistical difference in particle concentration between NPF and non-NPF days at the Modena and Ispra stations

	Size range	t-stastistic	P-value

Ispra station	10-25nm	7.5	4.9×10^{-10}
	25-50nm	4.9	7.1×10^{-6}
	50-100nm	1.5	0.11
	100-800nm	3.8	2×10^{-4}
Modena station	2-3nm	4.3	1.2×10^{-4}
	3-10nm	4.7	3.9×10^{-5}
	10-25nm	3	4.7×10^{-3}
	25-42nm	0.8	4.6×10^{-1}

Table S 3. The statistical difference between lockdown and after lockdown for the growth rate in the Modena station

	t-stastistic	P-value
<3 nm	0.9	0.3
3-7 nm	0.04	0.9
7-20 nm	0.1	0.9

Table S 4. The statistical difference between 2016-2019 and 2020 for the growth rate in the Ispra station

	t-stastistic	P-value
Pre lockdown	1.2	0.2
Lockdown	2.6	0.01
After lockdown	0.7	0.49

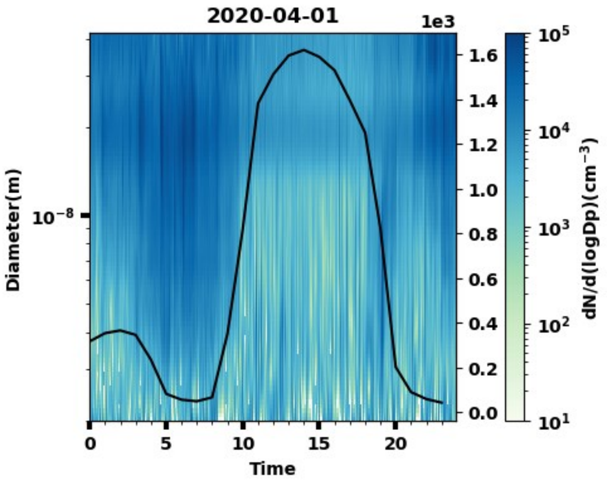

Figure S 4. The particle size distribution and boundary layer height in 01.04.2020.

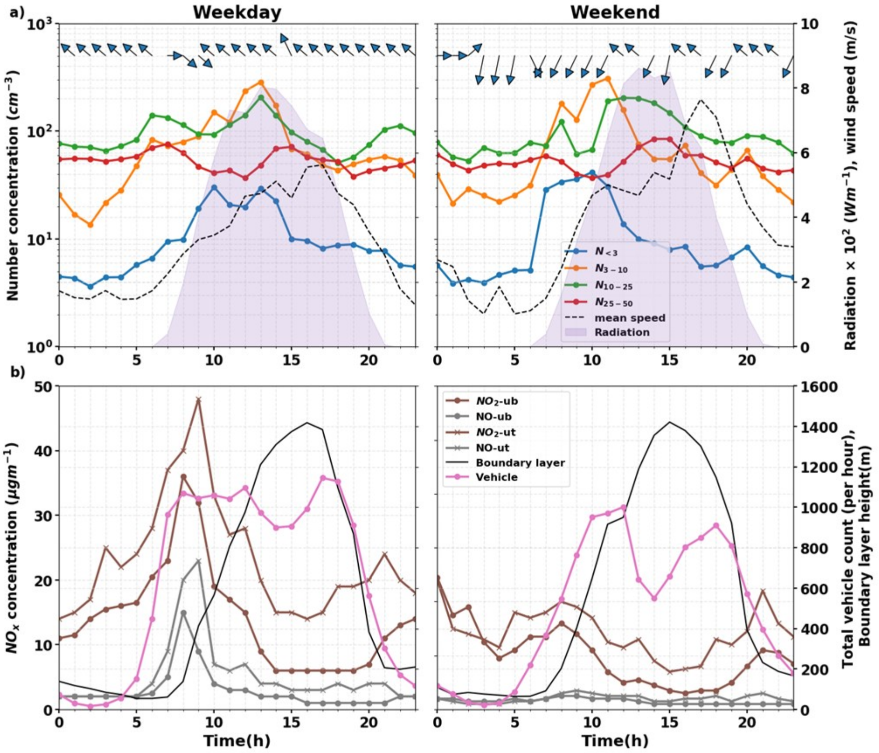

Figure S 5. a) Median diurnal variations after the lockdown period for the particles <50 nm, solar radiation (shaded area), wind speed (black dash line) and direction (blue arrow) in Modena. b) NO, NO_2 concentration from urban background (circle) and traffic (x) sites, total vehicle count (from the closest sensor) and boundary layer height.

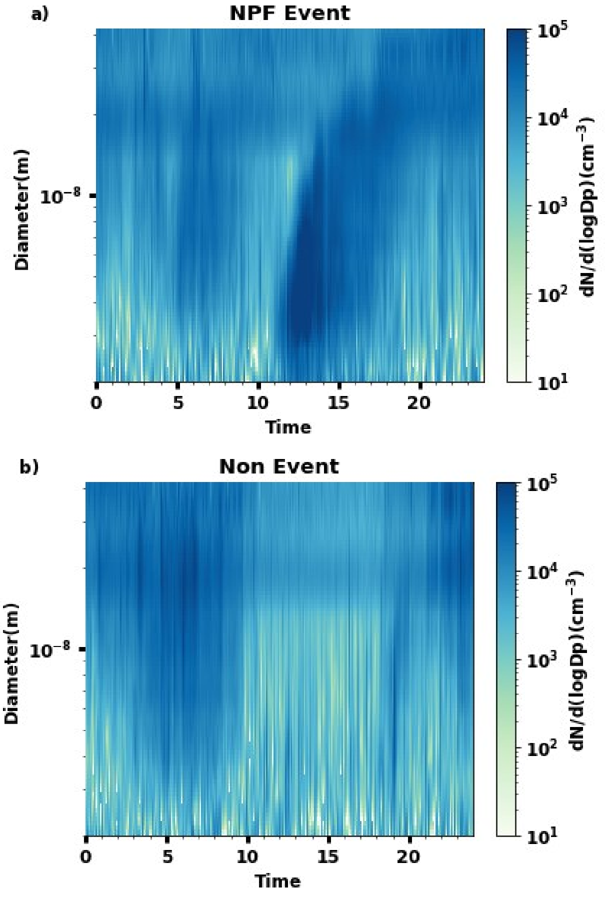

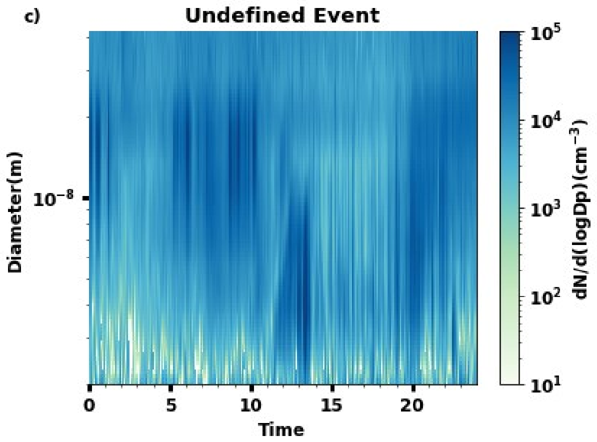

Figure S 6. Examples of the three different categories of new particle formation (NPF): NPF event (upper-left), non-event (upper-right), and an undefined day (bottom) at the Modena station.
